# The Association of Adipokines and Myokines in the Blood of Obese Children and Adolescents with Lipoprotein Lipase rs328 Gene Variants

**DOI:** 10.1155/2023/7392513

**Published:** 2023-10-20

**Authors:** Alexander V. Shestopalov, Vadim V. Davydov, Genrik T. Tumanyan, Elena D. Teplyakova, Tatiana P. Shkurat, Elena V. Mashkina, Mikhail A. Shkurat, Andrey M. Gaponov, Anastasia A. Sadova, Olga V. Borisenko, Sergey A. Roumiantsev

**Affiliations:** ^1^Pirogov Russian National Research Medical University, Moscow, Russia; ^2^The National Medical Research Center for Endocrinology, Moscow, Russia; ^3^Dmitry Rogachev National Medical Research Center of Pediatric Hematology, Oncology and Immunology, Moscow, Russia; ^4^The Rostov State Medical University, Rostov-on-Don, Russia; ^5^Southern Federal University, Rostov-on-Don, Russia; ^6^Limited Liability Company “Nauka”, Rostov-on-Don, Russia; ^7^V.A.Negovsky Research Institute of General Reanimatology, Federal Research and Clinical Center of Intensive Care Medicine and Rehabilitation, Moscow, Russia; ^8^Center of Digital and Translational Biomedicine (Center of Molecular Health), Moscow, Russia

## Abstract

Obesity develops largely due to genetic factors, with the genetic polymorphism of lipid metabolism enzymes being of particular importance. However, it is still unclear how the genetic variants of one of the key enzymes in lipid transport, lipoprotein lipase (LPL), are associated with the endocrine function of mesenchymal tissues in obesity. The current study was aimed at the investigation of the *LPL* rs328 gene variant association with adipokines and myokines levels, as well as lipid metabolism indices in the blood of children and adolescents of both genders with obesity. We found that *LPL* polymorphism rs328 is not characterized by the differences in the levels of hormones, adipokines, and myokines and in the blood of healthy children and adolescents; however, it significantly affects these indices during obesity in gender-dependent manner. The shifts in hormones, adipokines, and myokines manifest mostly in the obese individuals with Ser447Ser genotype rather than with 447Ter genotype. Obese boys homozygous for Ser447Ser have more elevated leptin levels than girls. They also demonstrate lower adiponectin, apelin, prolactin, and osteocrine levels than those in obese girls with the same genotype. The gender-based differences are less pronounced in individuals with 447Ter genotype than in the homozygotes for 447Ser. Thus, we conclude that the polymorphism rs328 of the lipoprotein lipase gene is accompanied by the changes in hormones, adipokines, and myokines levels in the blood of children and adolescents with obesity in gender-dependent manner.

## 1. Introduction

In recent years, the role of genetic factors in obesity development has been widely discussed [1]. There is abundant evidence pointing to the interconnection between this disease and the single nucleotide polymorphisms (SNPs) of genes coding for lipid metabolism enzymes [2–4]. The SNPs promote the formation of the enzymes with altered catalytic and regulatory properties [2–4], which may form the basis of triacylglycerol (TAG) metabolism impairment and subsequent accumulation of TAG in adipose tissue. At the same time, the interaction between SNPs and the production of biologically active compounds regulating lipid metabolism in patients with obesity is not studied thoroughly so far. Such an investigation is of particular interest in children and adolescents, i.e., at the first stage of the disease manifestation, as the timely diagnosis and therapy set-up may reduce the risk of cardiovascular complications with age [5, 6]. Moreover, the study of SNPs associations with hormone levels draws attention in regard to the development of new approaches for prognosis and prophylaxis of obesity at the early stages. This is especially urgent considering the increase in childhood obesity in recent decades [7, 8].

The key role in the metabolism of triacylglycerols in our body belongs to lipoprotein lipase (LPL). This enzyme is highly expressed in adipose tissue, myocytes, macrophages, and so on. The lipoprotein lipase gene (*LPL*) resides on chromosome 8 and codes for a precursor protein of 448 amino acid residues [2, 9, 10]. To date, hundreds of variants of the enzyme originating from the genetic polymorphisms in the 8p22 locus were identified. They underlay the peculiarities of lipid metabolism in individuals with the corresponding genotypes. For instance, rs1121923 and rs258 polymorphisms of LPL may have a protective function due to the changes in blood HDL and TAG levels. In this regard, these *LPL* gene variants are even recommended to include into a diagnostic panel for hyperlipidemia risk estimation [11].

The SNP rs328 in the *LPL* gene results from a point mutation, namely, the substitution of cytosine (C) to guanine (G) in the triplet coding for serine 447 in the polypeptide, leading to the formation of a premature stop-codon UGA (Ter). So, individuals with the 447Ter genotype have a truncated enzyme with higher catalytic activity [10, 11]. This isoform alters the blood levels of TAG, cholesterol, and HDLs; however, the data on these shifts are controversial [2, 11–14]. On the one hand, 447Ter is associated with the reduced risk of abdominal obesity [15], while on the other hand, carriers of the 447Ter genotype suffer from cardiovascular diseases more frequently [16].

At the same time, little is known about the role of the *LPL* SNPs in the changes of lipid metabolism indices and endocrine function of mesenchymal tissues in childhood obesity. Adipose tissue and muscles produce a wide spectrum of biologically active substrates. Adipokines, namely, leptin, adiponectin, resistin, and others, and myokines, such as irisin, adiponectin, and FGF-21 participate in the regulation of metabolism in both the corresponding tissues and the whole body. The alterations in their secretion are associated with the protective reactions and multiple impairments accompanying obesity (atherogenesis, damaging of tissues, and organs). Thus, in the present study, we focused on the association of the *LPL* polymorphism rs328 with the lipid metabolism indices and the levels of adipokines, myokines, and some other hormones in the blood of obese children and adolescents of both genders.

## 2. Materials and Methods

### 2.1. Participants

The study was conducted in 2019-2020 and included 200 Russian children and adolescents (boys and girls) aged 10–18 and attending school middle or senior classes. The mean age of healthy subjects was 11.9 ± 2.3 years, and the mean age of obese ones was 12.2 ± 2.0 years.

We carried out the following comparative analyses: (i) of healthy individuals (*n* = 100) and those with obesity (*n* = 100), (ii) of healthy boys (*n* = 61) and those with obesity (*n* = 48), (iii) of healthy girls (*n* = 39) and obese ones (*n* = 52), (iv) of healthy boys (*n* = 61) and healthy girls (*n* = 39), and (v) of boys and girls with obesity (*n* = 48 and *n* = 52, correspondingly). The participants were also grouped by genotype for *LPL* (rs328).

The participants addressed to Children's City Polyclinic 1 of Rostov-on-Don for a planned medical check-up. Out of them, 100 individuals made up a subject group, which included children and adolescents with previously diagnosed and managed alimentary constitutive obesity of different stages. As a control group, 100 healthy volunteers of the same age without obesity were examined.

Entry criteria for both groups were no antibiotics, probiotics, and prebiotics uptake 3 months before the set-up of the study, as well as the signed informed consent for participance in the study. Exit criteria for both groups were acute somatic diseases (chronic kidney, liver, or heart deficiency, and gastrointestinal diseases, namely, ulcerative colitis and Crohn's disease) and any acute condition. An additional criterion for the subject group was SDS body mass index (SDS BMI) >2.0 and the diagnosed alimentary constitutive obesity of I–III stage. The mean SDS BMI of healthy subjects was 0.76 ± 0.06, and the mean SDS BMI of obese ones was 2.6 ± 0.6.

The sampling was random with no advance computation of the size, and the study was single-center, one-stage, cluster-randomised.

### 2.2. Genotyping

The participants were genotyped for *LPL* polymorphism (rs328). Genomic DNA was extracted from blood leukocytes using the thermocoagulation method and the DNA-Express-Genetics kit (Lytech, Russia) according to the manufacturer's instructions. The participants were genotyped for *LPL* polymorphism (rs328) by allele-specific PCR using SNP-express (Lytech, Russia) reagents according the manufacturer's instructions. The run consisted of initial denaturation at 93°С for 1 min, followed by 35 cycles of 93°С for 10 s, 64°С for 15 s, 72°С for 20 s, and the final elongation at 72°C for 1 min. The amplicons were analyzed by horizontal gel-electrophoresis in 2.5% agarose gel.

### 2.3. Lipid Metabolism Indices

The biochemical indices, namely, total cholesterol (TC), high-density lipoprotein cholesterol (HDL-C), low-density lipoprotein cholesterol (LDL-C), very low-density lipoprotein cholesterol (VLDL-C), and triacylglycerols (TAG), were assessed using spectrophotometer Hitachi U-2900 (Japan) and corresponding commercial kits (Olvex Diagnosticum, Russia). Quantitative analyses of leptin, adiponectin, resistin, apelin, irisin, adipsin, myostatin, FGF21, osteocrine, oncostatin, and insulin were carried out using multiplex ELISA on Magpix (BioRad, USA) according to the manufacturer's instructions with Milliplex Human Adipokine Magnetic Bead Panel 1 and Human Adipokine Magnetic Bead Panel 2 (Merck, Germany). The concentrations of asprosin, prolactin, TTH, and *T*_4_ were determined using the enzyme immunoassay method. Glucose levels and the activity of alanine aminotransferase (ALT) and aspartic acid aminotransferase (AST) were also measured in blood serum.

HOMA-IR (fasting glucose to insulin ratio) was calculated as follows: fasting blood glucose (mmol/l) × insulin (*µ*U/l)/22.5. HOMA-IR above 2.7 was considered as insulin resistance.

The atherogenic index of plasma (AIP) was estimated according to the recommendations of NCEP ATPIII.

### 2.4. Statistics

R version 3.2 was used for statistical analysis (R Foundation for Statistical Computing, Vienna, Austria); the Shapiro–Wilk test was used as a test of normality. As the normal distribution was absent, comparative analyses were carried out using Mann–Whitney *U* test. The data were presented as medians and quartiles (25%, 75%), i.e., maximal and minimal values in a sample given in square brackets [Q1; Q4]. The differences were considered significant if *p* < 0.05.

### 2.5. Ethics

The study was approved by the local ethics committee of Pirogov Russian National Research Medical University (protocol No: 186, dated 26.06.2019). All subjects signed the informed consent for participance in the study.

## 3. Results and Discussion

According to the results (Tables 1 and 2), the Ser447Ser genotype is prevalent in the population under study. The stop-codon containing allele (447Ter) appears with almost the same frequency in boys and girls and makes up 13% and 10% of all of the subjects, correspondingly. Boys and girls with obesity have 447Ter genotype in 19% and 14% of cases, correspondingly.

Examination results for healthy and obese boys and adolescents with Lipoprotein Lipase rs328 Gene Variants.

Healthy boys and adolescents with both Ser447Ser genotype and 447Ter genotype do not show significant differences in the levels of adipokines, myokines, and biochemical blood indices. They also have no differences in SDS BMI. At the same time, as it can be observed from Table 1 that the individuals with Ser447Ser genotype have 41% and 72% lower blood levels of insulin and FGF21, correspondingly, compared to those with 447Ter genotype.

During obesity, both groups of boys with *LPL* polymorphism rs328 have pronouncedly increased SDS BMI and HOMA-IR. At the same time, they demonstrate elevated blood leptin and TTH than their healthy peers. It is worth to note that the rise in TTH level in boys with Ser447Ser genotype was two times lower than in 447Ter boys.

As it is demonstrated in Table 1, insulin, FGF21, irisin, and adipsin levels in the blood are higher, and blood prolactin level is lower in obese boys compared to those in healthy individuals. Also, 447Ter boys with obesity have 59% decreased apelin level compared to healthy peers with the same genotype.

The data obtained indicate pronounced changes of most of the indices under study in boys homozygous for 447Ser during obesity, which are not characteristic to the carriers of 447Ter allele. For both genotypes, we demonstrate that obesity is accompanied by elevated glucose levels and high ALT activity in the blood. In addition, 447Ter boys have increased AST activity, while Ser447Ser genotype is associated with declined HDL-C but increased VLDL-C, TAG, and AIP, compared to healthy boys from the corresponding control group.

Based on these data, we conclude that healthy boys with both genotypes for *LPL* rs328 do not demonstrate significant differences in the indices under study. Despite the distinctions in adipokines, myokines, and hormones in the blood of boys with obesity, the two genotypes do not differ in SDS BMI. Also, both 447Ser and 447Ter obese carriers develop insulin resistance (IR). Interestingly, Ser447Ser boys with obesity are characterized by hyperinsulinemia, unlike those with 447Ter in genotype.

Hyperleptinemia can be supposed to have a pivotal role in the pathogenesis of obesity in boys with different genetic variants of *LPL* SNP rs328. It serves as a background for hypothyroidism manifestation, which, in turn, leads to complications in the metabolism of lipids [17]. At this, Ser447Ser demonstrates characteristic changes in the blood levels of hormones and myokines with a clear adaptive tendency (e.g., decreased prolactin and, oppositely, elevated irisin, and FGF21). However, in spite of these adaptive changes, obese 447Ser homozygotes have pronounced alterations in the clinical indices indicating hyperlipidemia, atherogenesis, and other body malfunctioning processes.

Obese 447Ter boys do not have any changes in most of the indices under study, which is concordant with the published data [12, 14]. However, they have dramatically increased activity of transaminases (mostly, of AST) in the blood, which may evidence the malfunctioning of cardiovascular system and is also consistent with the current concepts [16].

The analysis of the data obtained allows to assume that obesity in boys with different *LPL* genetic variants of rs328 occurs due to similar pathogenetic mechanisms. Depending on the gene variant, they have different complications of the disease though. Obesity development in carriers of Ser447Ser genotype is accompanied by hyperlipidemia, tissue alterations, and atherosclerosis, while boys possessing 447Ter allele suffer from an increased risk of cardiovascular disease.

Examination results for healthy and obese girls and adolescents with Lipoprotein Lipase rs328 Gene Variants.

Healthy girls homozygous for 447Ser allele have 16% higher glucose and 20% lower cholesterol levels in the blood compared to those of the carriers of 447Ter allele (Table 2). Other clinical indices do not differ significantly in the two genotypes.

Obese girls with both *LPL* gene variants of rs328 have the same increased SDS BMI and leptin level with the simultaneous decline in blood asprosin. They also have significantly higher irisin levels compared to healthy girls with the corresponding genotypes for *LPL*.

As demonstrated in Table 2, Ser447Ser girls are characterized by upregulated levels of apelin, adipsin, osteocrine, FGF21, and insulin and downregulated resistin, unlike those with 447Ter genotype. Although blood adiponectin does not change significantly (*p* > 0.05), girls with Ser447Ser genotype have 2.1 times higher adiponectin than 447Ter girls.

Taking into account the biological role of the adipokines and myokines under study, their changing pattern in the blood is their protective role in obesity development. Thus, high leptin level promotes lipid catabolism and inhibits lipogenesis [18], and increased apelin lowers lipotoxicity and provides cardio- and neuroprotectivity together with activation of adipocytes proliferation and angiogenesis [19–22]. In its turn, upregulated irisin limits the energy imbalance in the body, decreases insulin resistance by increasing tissue sensitivity to insulin, promotes *β*-cell proliferation in the islets of Langerhans, prevents cardiovascular disease progression, inhibits the production of proinflammatory cytokines by adipose tissue, acts as a messenger of metabolic communication between the tissues, and so on [23–27]. In addition, the decreased asprosin level together with leptin predetermines the anorexigenic effect.

The absence of a statistically significant (*p* > 0.05) increase in the level of blood insulin in obese 447Ter girls draws attention. Because of that, they may have developed only a clear tendency to IR, unlike those with Ser447Ser genotype. Moreover, 447Ter genotype is characterized by decreased prolactin concentration in the blood during obesity.

The changes in adipokines, myokines, and blood hormones revealed in obese girls homozygous for 447Ser are accompanied by elevated VLDL-C and TAG and decreased HDL-C, which is not observed in girls 447Ter-carriers with obesity. However, the latter have 23% decreased total cholesterol compared to that in healthy individuals with the same genotype.

The study demonstrates that girls with different *LPL* gene variants for rs328 have the same SDS BMI in obesity. Their disease is accompanied by hyperleptinemia, which apparently serves as one of the key factors in the pathogenesis of obesity. However, in contrast to boys with *LPL* SNP rs328, high leptin level in the blood is not accompanied by hypothyroidism symptoms exacerbating obesity.

Despite the particular similarities in the changes of the indices under study, girls with different genotypes for *LPL* gene variant rs328 show their own peculiarities in the changes of hormones, adipokines, and myokines levels in the blood during obesity. For instance, Ser447Ser girls are characterized by a higher lability in the indices measured. Moreover, the changes observed are either compensatory or IR-derived.

Thus, we consider increased irisin and adipsin and decreased resistin and asprosin to be compensatory changes in the carriers of the two 447Ser alleles with obesity. The compensation in 447Ter girls manifests as decreased asprosin and increased irisin concentrations in the blood. It is worth to note that the rise of the latter is less intense in the carriers of 447Ter allele compared to Ser447Ser individuals. At the same time, 447Ter girls with obesity demonstrate a dramatic decrease in adiponectin and prolactin compared to 447Ser homozygotes. The latter have elevated osteocrine concentrations, which plays a vital role in the protection of cardiovascular system and central nervous system during obesity [28].

We conclude that female individuals with different SNP variants for *LPL* develop their particular protective mechanisms during obesity. As a consequence, the complications of the disease may also differ. As appears from the clinical data (Table 2), 447Ter girls with obesity have no hyperlipidemia manifestations, which are characteristic for obese girls with Ser447Ser genotype. Moreover, they have decreased total cholesterol in the blood.

Another distinctive feature of obesity development in girls with Ser447Ser genotype is IR, which is not characteristic for obese girls who carry 447Ter allele. Possibly, IR accompanied by hyperinsulinemia leads to increased adipsin and apelin levels and decreased asprosin in the blood. Insulin resistance can also be associated with their typical change in blood adiponectin concentration.

The data analysis allows to conclude that girls with different *LPL* gene variants for rs328 have their own peculiarities in the production of hormones, adipokines, and myokines, which underly the protective function. They all aimed at the limitation of white adipose tissue mass, decrease in lipotoxicity, inhibition of proinflammatory cytokines synthesis by adipocytes, and protection of cardiovascular and central nervous system. However, even at this, girls homozygous for 447Ser allele develop hyperlipidemia and insulin resistance typical for obesity. Despite its probable compensatory function for lipogenesis limitation at the first stage of the disease, IR makes a threat of diabetes type 2 development in girls with age. 447Ter genotype is characterized by the resistance to changes in blood lipids, IR, and tissue damage, which was reported before [12, 14].

Gender-based differences in the blood level of adipokines, myokines, and hormones of children with Lipoprotein Lipase rs328 gene variants and its significance in the development obesity and concomitant disorders of metabolism.

Our study reveals the association between the rs328 genetic variant of lipoprotein lipase and blood levels of particular hormones, adipokines, and myokines in children and adolescents with obesity. Gender-derived associations are also noticed. Obese boys with Ser447Ser genotype demonstrate 1.7 times higher leptin levels compared to obese girls with the same genotype. Adiponectin, apelin, prolactin, and osteocrine levels are 50%, 85%, 25%, and 29%, correspondingly, lower in boys with obesity homozygous for 447Ser than those in the corresponding group of obese girls.

The estimation of a possible role of these differences indicates that male individuals with Ser447Ser genotype have reduced adaptive changes in the production of hormones, adipokines, and myokines during obesity.

One of the most typical for obesity gender-based differences in the blood level of adipokines is the change of apelin level. Boys with different genotypes for rs328 of *LPL* have several times lower levels of this adipokine compared to girls with different genotypes. Considering its possible role in the regulation of cell differentiation [21, 22], one can suggest that the upregulation of apelin favors white adipocytes proliferation. This, in turn, predetermines the features of obesity development and manifestation in boys and girls [29].

Based on the above said, one can come to a conclusion that pathogenesis of obesity in boys with Ser447Ser genotype for lipoprotein lipase (rs328) is associated with limited adaptation processes in the body. Subsequent upon this, they have 15% higher SDS BMI compared to girls from the corresponding group. Moreover, in contrast to girls, they have more pronounced tissue alteration processes and atherogenesis.

Gender-based differences are less distinct in the carriers of 447Ter genotype than in 447Ser homozygotes. However, even at this obese 447Ter boys still have signs of intense tissue damage and alteration.

In sum, healthy children and adolescents with genetic variants of rs328 do not show significant differences in the blood levels of hormones, adipokines, and myokines under study. During obesity, the changes in them are more evident in boys and girls with Ser447Ser genotype than with 447Ter one.

Gender-associated changes in the blood indices of participants with obesity depend on the genotype. Boys homozygous for 447Ser have more clearly increased leptin levels than girls, while adiponectin, apelin, prolactin, and osteocrine levels in their blood appear to be significantly lower compared to those of obese girls. As already stated, gender-derived differences are feeble in 447Ter genotype compared to 447Ser homozygotes.

The nature of the changes in adipokines, myokines, and some hormones in the blood of boys with Ser447Ser genotype indicates limited protective reactions and adaptation of metabolism during obesity. Possibly, this may serve as one of the causes of the increased adipose tissue mass, compared to obese girls, as well as hyperlipidemia development, tissue alteration, and atherogenesis. Girls with the rs328 genetic variant of *LPL* have clear changes in the production of hormones, adipokines, and myokines in the blood, which have protective properties during obesity. However, obese girls homozygous for 447Ser have IR and hyperlipidemia, which are not typical for girls with 447Ter allele.

The data obtained reflect the contribution of LPL SNP rs328 to the changes of the blood levels of hormones, adipokines, and myokines in children and adolescents with obesity in gender-dependent manner.

These changes become of particular importance in the obesity pathogenesis, for they cause the peculiarities in the development of the comorbidities and tissue damage. This must be taken into consideration during the treatment of obesity in patients of the given age, as well as during the prognosis of the disease and the complications development.

The specific mechanisms of the interconnection between the LPL polymorphism and the blood levels of individual adipokines and myokines in children with the different genetic variants remain unknown. Their investigation shows considerable promise in the development of new pathogenetic approaches for the treatment and prophylaxis of obesity in children and adolescents. Our upcoming studies will be dedicated to solving these questions.

## 4. Conclusions

1. Healthy children with different *LPL* gene variants for rs328 do not demonstrate any significant differences in the blood levels of hormones, adipokines, and myokines.
2. SNP rs328 of *LPL* gene significantly affects the blood levels of hormones, adipokines, and myokines in children and adolescents with obesity in gender-dependent manner. These shifts may play an important role in the development of obesity and the accompanying disorders (dyslipidemia, atherogenesis, and tissue alterations), which affect the prognosis of the disease.
3. The changes in the blood levels of hormones, adipokines, and myokines are more pronounced in obese children (boys and girls) with Ser447Ser genotype than in the carriers of 447Ter allele.
4. Boys homozygous for 447Ser allele have more elevated leptin, while adiponectin, apelin, prolactin, and osteocrine levels in their blood are significantly lower compared to those of obese girls with the same genotype. At this, the latter develop the protective reactions during obesity.
5. Gender-based differences are less pronounced in the carriers of 447Ter allele than in Ser447Ser homozygotes.
6. The genotype of the patients with the polymorphism rs328 of *LPL* must be taken into account during the prognosis of obesity and the development of its complications in children and adolescents.

## Figures and Tables

**Table 1:** The levels of adipokines, myokines, hormones, and biochemical indices in the blood of healthy and obese boys with different genotypes for LPL (rs328).

Index	Control group (healthy boys)		Subject group (boys with obesity)	
Genotype	Ser447Ser	447Ter	Ser447Ser	447Ter
SDS BMI	0.9 (0.4; 1.3)	0.8 (0.3; 1.4)	2.73 (2.44; 3.21) *p*_1_ < 0.001 *p*_9_ < 0.001	2.64 (2.34; 2.88) *p*_2_ < 0.001
Leptin (pg/ml)	2.3 (1.3; 4.2)	2.2 (1.5; 3.1)	19.0 (15.3; 19.2) *p*_1_ < 0.001 *p*_9_ = 0.003	19.0 (19.0; 22.6) *p*_2_ < 0.001
Adiponectin (*µ*g/ml)	216 (167; 428)	293 (201; 406)	195 (156; 262) *p*_9_ < 0.001	310 (167; 388)
Resistin (ng/ml)	26.8 (14.1; 101.9)	38.6 (21.3; 114.3)	47.2 (28.9; 80.6)	42.2 (26.9; 109.6)
Apelin (pg/ml)	36 (21; 53)	40 (36; 44)	22.3 (16.3; 133.2) *p*_9_ = 0.001	16.3 (16.3; 21.1) *p*_2_ = 0.011 *p*_10_ = 0.001
Asprosin (ng/ml)	0.4 (0; 1.1)	0.6 (0; 0.6)	0.2 (0; 0.5)	0.3 (0; 0.4)
TTH (mU/l)	2.1 (1.4; 2.7)	2.5 (1.9; 2.7)	2.9 (2.2; 4.1) *p*_1_ < 0.001 *p*_7_ = 0.03	4.2 (4.0; 5.2) *p*_2_ = 0.001
Т_4_ (pmol/l)	11.9 (10.9; 13.2)	11.6 (106; 11.8)	12.2 (11.2; 13.1)	11.8 (11.4; 12.1)
Prolactin (mU/l)	148 (111; 185)	181 (135; 228)	124 (84.9; 151) *p*_1_ = 0.035 *p*_9_ = 0.001	133 (75.7; 187)
Insulin (ng/ml)	10.2 (8.9; 10.1) *p*_5_ = 0.007	17.4 (15.7; 22.2)	30.1 (18.9; 41.3) *p*_1_ < 0.001	24.3 (21.5; 34.0)
НОМА-IR	1.7 (1.2; 2.4)	2.5 (2.0; 3.4)	5.6 (3.4; 7.6) *p*_1_ < 0.001	4.8 (3.8; 5.8) *p*_2_ = 0.027
FGF21 (ng/ml)	11 (10; 18) *p*_5_ = 0.005	30.5 (18.8; 44.8)	18.4 (10.3; 47.6) *p*_1_ = 0.007	18.4 (9.5; 30)
Irisin (ng/ml)	200 (96; 244)	244 (96; 244)	186.9 (98.2; 293) *p*_1_ = 0.025	244.1 (92.5; 29)
Adipsin (*µ*g/ml)	2.5 (1.7; 3.8)	3.5 (2.0; 4.1)	4.3 (3.0; 4.0) *p*_1_ = 0.003	4.3 (2.6; 4.6)
Myostatin (ng/ml)	337 (288; 488)	488 (438; 488)	387.3 (236; 488)	361.2 (274; 362)
Oncostatin (pg/ml)	10 (7; 16)	19 (10.5; 26.5)	9 (7; 17) *p*_9_ = 0.005	12 (7; 17)
Osteocrine (ng/ml)	88 (58; 108)	82.5 (68.3; 97.3)	66.7 (52.1; 96.2) *p*_7_ = 0.049 *p*_9_ = 0.001	104.3 (92.5; 122)

*Biochemical indices*				
Glucose (mmol/l)	3.5 (3.0; 3.9)	2.9 (2.9; 3.4)	4.4 (3.7; 4.6) *p*_1_ < 0.001	4.4 (3.9; 4.6) *p*_2_ = 0.034
ALT (U/l)	13.8 (11.1; 18)	10.6 (9.7; 15.9)	21.3 (13.3; 26.8) *p*_1_ = 0.007 *p*_9_ = 0.013	29.1 (17.4; 40.2) *p*_2_ = 0.028 *p*_10_ = 0.026
AST (U/l)	24 (21.9; 27.6)	19.6 (16.2; 24.2)	24.9 (22.3; 28.9) *p*_9_ = 0.005	34.1 (28.4; 34.8) *p*_2_ = 0.011 *p*_10_ = 0.021
Total cholesterol (TC) (mmol/l)	3.9 (3.5; 4.7)	3.8 (3.4; 4.2)	3.9 (3.7; 4.2)	4.3 (3.8; 4.6)
HDL-cholesterol (mmol/l)	1.3 (1.2; 1.5)	1.2 (0.9; 1.3)	1.2 (1.0; 1.2) *p*_1_ = 0.002	1.1 (1.0; 1.2)
LDL-cholesterol (mmol/l)	2.4 (1.9; 3.0)	2.1 (1.4; 2.3)	2.1 (1.8; 2.4)	2.4 (1.8; 2.8)
VLDL-cholesterol (mmol/l)	0.3 (0.2; 0.5)	0.5 (0.3; 0.8)	0.6 (0.5; 0.7) *p*_1_ < 0.001	0.7 (0.6; 0.8)
TAG (mmol/l)	0.7 (0.5; 1.1)	0.9 (0.7; 1.5)	1.2 (1; 1.5) *p*_1_ < 0.001	1.4 (1.2; 1.6)
Atherogenic index of plasma (AIP)	1.9 (1.6; 2.5)	2.5 (1.9; 3.0)	2.4 (1.9; 3.1) *p*_1_ = 0.016	2.4 (2.1; 2.8)

Comment: The data are presented as median [Q1; Q4]. The table contains statistically significant *p* values (*p* < 0.05) between the compared groups: *p*_1_—healthy Ser/Ser boys compared to obese Ser/Ser boys; *p*_2_—healthy 447Ter boys compared to obese 447Ter boys; *p*_5_—healthy Ser/Ser boys compared to healthy 447Ter boys; *p*_7_—obese Ser/Ser boys compared to obese 447Ter boys; *p*_9_—obese Ser/Ser boys compared to obese Ser/Ser girls; *p*_10_—obese 447Ter boys compared to obese 447Ter girls.

**Table 2:** The levels of adipokines, myokines, hormones, and biochemical indices in the blood of healthy and obese girls with different genotypes for LPL (rs328).

Index	Control group (healthy girls)		Subject group (girls with obesity)	
Genotype	Ser447Ser	447Ter	Ser447Ser	447Ter
SDS BMI	0.69 (0.27; 0.9)	0.11 (0.1; 0.5)	2.37 (2.13; 2.61) *p*_3_ < 0.001	2.6 (2.14; 2.71) *p*_4_ < 0.001
Leptin (pg/ml)	5.0 (3.1; 6.2)	4.5 (3.6; 5.5)	14.6 (12.0; 218.3) *p*_3_ < 0.001	14.0 (6.5; 19.1) *p*_4_ = 0.034
Adiponectin (*µ*g/ml)	275 (172; 480)	291 (178; 415)	393 (245; 479) *p*_8_ = 0.007	186 (142; 214)
Resistin (ng/ml)	97.4 (40.9; 142)	80.1 (41.2; 126.5)	43.9 (27.1; 81.1) *p*_3_ = 0.014	43.3 (22.7; 126.8)
Apelin (pg/ml)	31.6 (21.1; 43.9)	118.8 (79.8; 145)	153 (118.8; 225) *p*_3_ < 0.001	228 (140.4; 310)
Asprosin (ng/ml)	0.5 (0; 0.9)	0.,6 (0.6; 0.8)	0.2 (0; 0.5) *p*_3_ = 0.018	0 (0; 0.1) *p*_4_ = 0.006
TTH (mU/l)	2.5 (1.8; 3.4)	1.6 (1.5; 3.2)	3.0 (2.5; 4.2)	3.4 (2.9; 4.8)
Т_4_ (pmol/l)	11.4 (10.6; 12.5)	10.9 (10.2; 11.5)	11.9 (10.8; 12.3) *p*_3_ = 0.004	10.9 (10.9; 11.4)
Prolactin (mU/l)	229 (162; 311)	176 (162.8; 236)	165 (113; 243.5) *p*_8_ = 0.011	96.2 (86.1; 117.6) *p*_4_ = 0.038
Insulin (ng/ml)	14.6 (13.2; 18.6)	24.7 (15.1; 32.8)	24.7 (19.5; 30.8) *p*_3_ < 0.001	26.4 (18.9; 45.7)
НОМА-IR	2.3 (1.9; 3.3)	2.7 (2.1; 3.5)	4.1 (3.3; 6.1) *p*_3_ < 0.001	3.6 (3.1; 8.5)
FGF21 (ng/ml)	10 (5.2; 14.5)	5.5 (5.2; 15.5)	18 (6; 24) *p*_3_ = 0.008	11 (10.5; 24.5)
Irisin (ng/ml)	96 (52.4; 109.7)	79.5 (63.4; 100.8)	244.1 (195; 360) *p*_3_ < 0.001	244 (218.5; 402) *p*_4_ = 0.008
Adipsin (*µ*g/ml)	2.4 (1.5; 3.9)	3.4 (2.4; 4.9)	3.1 (2.3; 4.3) *p*_3_ = 0.045	3.7 (2.7; 4.0)
Myostatin (ng/ml)	488.3 (336.9; 488.3)	488 (450.2; 488.1)	454 (374; 488)	361 (211; 374)
Oncostatin (pg/ml)	12.9 (9.2; 16.4)	15.8 (13.9; 21.1)	14 (10; 21.5)	15 (13; 38)
Osteocrine (ng/ml)	77.1 (58.9; 96.5)	86.8 (65.5; 131.9)	94 (80; 117.5) *p*_3_ = 0.004	103 (80; 128)

*Biochemical indices*				
Glucose (mmol/l)	3.6 (3.2; 3.9) *p*_6_ = 0.029	3.1 (2.8; 3.2)	4.2 (3.5; 4.4) *p*_3_ = 0.025	3.9 (3.5; 4.1)
ALT (U/l)	13.4 (11.1; 16.3)	11.6 (10.5; 23.1)	15.2 (12.3; 17.8)	13 (11.9; 14.1)
AST (U/l)	20.3 (18.6; 26.2)	22.2 (20; 24.7)	21.4 (19.7; 24.9)	21.2 (18.7; 24.8)
Total cholesterol (TC) (mmol/l)	4.1 (3.7; 4.6) *p*_6_ = 0.012	5.0 (4.5; 5.5)	4.0 (3.6; 4.2)	3.9 (3.7; 4.3) *p*_4_ = 0.014
HDL-cholesterol (mmol/l)	1.3 (1.1; 1.5)	1.3 (1.1; 1.7)	1.3 (1.0; 1.4) *p*_3_ = 0.013	1.1 (0.9; 1.6)
LDL-cholesterol (mmol/l)	2.3 (1.8; 2.6)	2.6 (2.4; 2.9)	2.2 (1.9; 2.4)	2.1 (1.7; 2.4)
VLDL-cholesterol (mmol/l)	0.4 (0.3; 0.6)	0.8 (0.5; 1.2)	0.5 (0.4; 0.8) *p*_3_ = 0.009	0.6 (0.4; 0.8)
TAG (mmol/l)	0.8 (0.6; 1.1)	1.6 (1.0; 2.3)	1.1 (0.9; 1.5) *p*_3_ = 0.007	1.1 (0.7; 1.6)
Atherogenic index of plasma (AIP)	2.0 (1.6; 2.6)	2.9 (2.4; 3.5)	2.5 (1.9; 2.8)	2.0 (1.8; 3.1)

Comment: The data are presented as median [Q1; Q4]. The table contains statistically significant *p* values (*p* < 0.05) between the compared groups: *p*_3_—healthy Ser/Ser girls compared to obese Ser/Ser girls; *p*_4_—healthy 447Ter girls compared to obese 447Ter girls; *p*_6_—healthy Ser/Ser girls compared to healthy 447Ter girls; *p*_8_—obese Ser/Ser girls compared to obese 447Ter girls;

## Data Availability

The data used to support the study are available from the corresponding author upon request.
